# Comparing a few SNP calling algorithms using low-coverage sequencing data

**DOI:** 10.1186/1471-2105-14-274

**Published:** 2013-09-17

**Authors:** Xiaoqing Yu, Shuying Sun

**Affiliations:** 1 Department of Epidemiology and Biostatistics, Case Western Reserve University, Cleveland, Ohio 44106, USA; 2 Department of Mathematics, Texas State University, San Marcos, Texas 78666, USA

**Keywords:** Next generation sequencing, SNP calling, Low-coverage, Single-sample, SOAPsnp, Atlas-SNP2, SAMtools, GATK

## Abstract

**Background:**

Many Single Nucleotide Polymorphism (SNP) calling programs have been developed to identify Single Nucleotide Variations (SNVs) in next-generation sequencing (NGS) data. However, low sequencing coverage presents challenges to accurate SNV identification, especially in single-sample data. Moreover, commonly used SNP calling programs usually include several metrics in their output files for each potential SNP. These metrics are highly correlated in complex patterns, making it extremely difficult to select SNPs for further experimental validations.

**Results:**

To explore solutions to the above challenges, we compare the performance of four SNP calling algorithm, SOAPsnp, Atlas-SNP2, SAMtools, and GATK, in a low-coverage single-sample sequencing dataset. Without any post-output filtering, SOAPsnp calls more SNVs than the other programs since it has fewer internal filtering criteria. Atlas-SNP2 has stringent internal filtering criteria; thus it reports the least number of SNVs. The numbers of SNVs called by GATK and SAMtools fall between SOAPsnp and Atlas-SNP2. Moreover, we explore the values of key metrics related to SNVs’ quality in each algorithm and use them as post-output filtering criteria to filter out low quality SNVs. Under different coverage cutoff values, we compare four algorithms and calculate the empirical positive calling rate and sensitivity. Our results show that: 1) the overall agreement of the four calling algorithms is low, especially in non-dbSNPs; 2) the agreement of the four algorithms is similar when using different coverage cutoffs, except that the non-dbSNPs agreement level tends to increase slightly with increasing coverage; 3) SOAPsnp, SAMtools, and GATK have a higher empirical calling rate for dbSNPs compared to non-dbSNPs; and 4) overall, GATK and Atlas-SNP2 have a relatively higher positive calling rate and sensitivity, but GATK calls more SNVs.

**Conclusions:**

Our results show that the agreement between different calling algorithms is relatively low. Thus, more caution should be used in choosing algorithms, setting filtering parameters, and designing validation studies. For reliable SNV calling results, we recommend that users employ more than one algorithm and use metrics related to calling quality and coverage as filtering criteria.

## Background

SNPs, which make up over 90% of all human genetic variations [1], contribute to phenotype differences and disease risk. Due to their high frequency and binary variation patterns, SNPs have been widely used as generic markers in disease association studies to identify genes associated with both monogenic [2] and complex diseases, such as diabetes [3-6], autoimmune diseases [7-9], cancers [10,11], and Alzheimer’s disease [12,13]. SNPs also serve as popular molecular markers in pharmacogenomic studies to understand inter-individual differences in response to treatments [14,15]. Therefore, it is essential to obtain accurate SNP information through advanced methods, such as, high throughput next-generation sequencing (NGS) technologies.

NGS technologies (e.g., the Solexa/Illumina sequencer, 454/Roche system, and SOLiD/ABI system) have been widely used in the last several years [16]. A single sequencing run by an NGS platform can generate data in the gigabase-pair scale, which usually contains millions and even hundreds of millions of sequencing reads. This high throughput makes NGS technologies more suitable for SNV identification compared to traditional technologies. However, challenges are also present. To produce such an enormous amount of data, multiple sequencing procedures (e.g., template amplification, florescent intensity detection, and base calling) are involved in NGS technologies [17]. As a result, artifacts can be introduced by both systematic and random errors. These errors include mishandled templates, PCR amplification bias, and florescence noises. Since the SNV detection relies on the identification of polymorphisms at the level of individual base pairs, any sequencing error can lead to an incorrect SNP identification. Furthermore, other genetic variations (e.g., copy number variation, insertion, deletion, inversion, and rearrangements) make accurate SNP calling even more difficult.

In order to identify SNVs using NGS data, various SNP calling programs have been subsequently developed [18-33]. For a general survey on SNP calling programs, please check the review paper by Pabinger *et al*. [34]. These programs serve as useful tools to detect SNPs from high throughput sequencing data and greatly extend the scale and resolution of sequencing technology applications. Our preliminary work has shown that for sequencing datasets that have high coverage and are of high quality, SNP calling programs can perform similarly [35]. However, when the coverage level is low in a sequencing dataset, it is challenging to accurately call SNVs [36]. Moreover, commonly used SNP calling programs (e.g., SOAPsnp [19], Atlas-SNP2 [20], SAMtools [37], and GATK [27,38]) all include different metrics for each potential SNP in their output files. These metrics are highly correlated in complex patterns, which make it challenging to select SNPs that are used for further experimental validations. In order to accurately detect SNPs from a low-coverage sequencing dataset, effective solutions have been in great demand. Some studies have shown that incorporating haplotype information and other pooled information can help in identifying SNPs in multiple-sample datasets [36,39,40]. However, many pilot studies have a small sample size (e.g., one or two samples), so the multiple-sample methods cannot be applied. Although the difficulty of SNP calling using single-sample low-coverage sequencing data has been recognized, it is still unclear how well different SNP calling algorithms perform and how to choose reliable SNPs from their results.

In this paper, we have conducted a systematic analysis using a single-sample low-coverage dataset to compare the performance of four commonly used SNP calling algorithms: SOAPsnp, Atlas-SNP2, SAMtools, and Unified Genotyper (UGT) in GATK. We have also explored the filtering choice based on the metrics reported in the output files of these algorithms. First, we improve the quality of the raw sequencing data by trimming off the low quality ends for reads in the data, then call SNVs using the four algorithms on these trimmed sequencing reads. We compare the SNV calling results from the four algorithms without using any post-output filters. Second, we explore the values of a few key metrics related to SNVs’ quality in each algorithm and use them as the post-output filtering criteria to filter out low quality SNVs. Third, we choose several cutoff values for the coverage of called SNVs in order to increase the agreement among the four algorithms. With the above analysis procedure, our goal is to offer insights for efficient and accurate SNV calling using a single-sample low-coverage sequencing dataset.

## Methods

### Part I Reviewing the key features of SNP calling algorithms

#### *Preprocessing steps of different SNP calling algorithms*

Alignment (i.e., mapping the reads back to a reference genome) is a fundamental and crucial step of any NGS data analysis, including SNP calling. In order to eliminate the possible sources of calling errors in the alignment results, almost all SNP calling algorithms incorporate certain processing steps as shown in Table 1. In this section, we review these steps one by one.

1) In order to deal with duplicate reads that may be generated during PCR, Atlas-SNP2, SAMtools, and GATK remove all the reads with the same start location in the initial alignment, except the one that has the best alignment quality. In contrast, instead of removing the duplicate reads, SOAPsnp sets a penalty to reduce the impact of these duplications.

2) In order to deal with reads that are aligned to multiple locations on the genome, SOAPsnp only takes into account the uniquely aligned reads, i.e., reads with only one best hit (the alignment with the least number of mismatches). Atlas-SNP2, GATK, and SAMtools do not have a specific strategy to deal with the multiple-hit issue, instead these calling programs accept all hits that the alignment results provide.

3) In order to make sure the sequencing quality of each read reflects the true sequencing error rate, SOAPsnp, SAMtools, and GATK recalibrate the raw sequencing quality scores generated by NGS platforms. Key factors, such as raw quality scores, sequencing cycles, and allele types, are all considered.

4) In order to deal with the presence of indels, both SAMtools and GATK include a realignment step to ensure accurate variant detection. In particular, GATK constructs the haplotype that could best represent the suspicious regions and realigns these regions appropriately according to this best haplotype. In contrast, SOAPsnp and Atlas-SNP2 do not utilize a specific indel realignment algorithm. SOAPsnp authors have conducted a simulation using a set of simulated data with 10,000 indels, and have shown that only 0.6% of reads containing indels are misaligned, and only 0.03% of those incorrect SNPs are retained in the final SNP calling output after routine processes including pre-filtering and genotype determination.

**Table 1:** Preprocessing steps in each of the four algorithms

	**SOAPsnp**	**Atlas-SNP2**	**SAMtools**	**GATK**
**Version**	1.03	1.2	1.1.18	1.6
**Format of aligned reads**	SOAP output	SAM/BAM	BAM	SAM/BAM
**Duplicate reads**	Penalty	Remove using Atlas-SNP-mapper	Removed	Remove using picard [41]
**Reads with multiple-hit**	Remove	Keep all hits	Keep all hits	Keep all hits
**Quality recalibration**	Yes	No	Yes	Yes
**Realignment**	No	No	Yes	Yes

#### *SNP calling*

In order to identify novel SNPs using sequencing reads and their quality scores, all four SNP calling programs apply the Bayesian method. SOAPsnp, SAMtools, and GATK-UGT compute the posterior probability for each possible genotype, and then choose the genotype with the highest probability (*P*_*H*_) as the consensus genotype. A SNP is called at a specific position if its consensus genotype is different from the reference. As a result, for both SOAPsnp and SAMtools, a *phred*-like consensus quality score, representing the accuracy of the SNP calling, is calculated as − 10 log _10_[1 − *P*_*H*_]. Different from the other three algorithms, Atlas-SNP2 calculates the posterior probabilities for each variant allele instead of the genotype, and the genotype is determined afterwards according to the ratio of the number of reads covering the reference and the number of reads covering the most likely variant. Depending on the Bayesian framework that each SNP calling program uses, different sets of metrics can be considered in SNP calling procedures (Table 2). Several common parameters are often considered by most calling programs (e.g., quality scores, sequencing cycles, and allele types). There are also some parameters specifically adopted by each algorithm. In particular, Atlas-SNP2 considers several unique metrics: 1) whether the allele is involved in a multi-nucleotide polymorphism (MNP) event; 2) whether the allele is a “swap-base”, defined as the situation in which two adjacent mismatches invert their nucleotides respective to the reference; 3) whether the allele passes the neighboring quality standard (NQS), which means that the quality score of the variant allele should be higher than 20, and the quality score of each of the five flanking bases on both sides should be higher than 15; and 4) whether the variant allele coverage is at least 3. SAMtools incorporates two unique metrics, base dependency and strand independency. The former accounts for the correlation between bases, while the latter assumes that reads from different strands are more likely to have independent error probabilities.

**Table 2:** Metrics considered in calling SNPs by each of the four algorithms

	**SOAPsnp**	**Atlas-SNP2**	**SAMtools**	**GATK-UGT**
**Quality score**	Recalibrated	Raw	Recalibrated	Recalibrated
**Machine cycle**	Yes	Yes	No	Yes
**Allele type**	Yes	No	No	Yes
**Duplication level**	Penalty in quality score	No	No	No
**Swap-base**	No	Yes	No	No
**MNP events**	No	Yes	No	No
**NQS**	No	Yes	No	No
**Coverage variation**	No	Yes	No	No
**Base dependency**	Yes	No	Yes	No
**Strand independency**	No	NO	Yes	No

#### *Built-in filters*

After obtaining the raw genotypes or variant alleles, several internal filters are used by Atlas-SNP2, SAMtools and GATK-UGT to further identify potential SNPs (Table 3). For example, Atlas-SNP2 allows users to set up a cutoff value for posterior probability to get a customized list of potential variants among those putative variant alleles. The genotyping results are given in a variant call format (VCF) output file and several criteria are applied to determine the final genotypes:

(1) Both strands are required to be supported by variant alleles.

(2) Cutoff values for the percentage of variant reads are set to determine homozygous or heterozygous genotypes. In particular, at a specific locus, if less than 10% of the total reads support the variant allele, the genotype is determined to be a homozygous reference for this locus; if the percentage of variant reads is between 10% and 90%, a heterozygous genotype is assigned to this locus; if the percentage of variant reads is higher than 90%, this locus is determined as a homozygous variant.

(3) A binomial test is employed to estimate the genotype qualities, and gives a posterior probability to indicate how confident the algorithm is in calling this position as a variant.

**Table 3:** Criteria for calling a SNP in each of the four algorithms

	**SOAPsnp**	**Atlas-SNP2**	**SAMtools**	**GATK -UGT**
**Quality score**	No	Yes	Yes	Yes
**Strand bias**	No	Both strands must be covered by variant allele	Yes	Yes
**Coverage limits**	No	variant allele coverage ≥ 3 upper limits for coverage	Yes	No
**Variant reads percentage**	No	Heterozygous: ≥ 10% Homozygous variant: ≥ 90%	No	No
**SNP Location**	No	No	No	No

Similar to Atlas-SNP2, SAMtools and UGT also produce SNP calling results in VCF output. Therefore, the internal filtering criteria of VCF are incorporated in GATK-UGT and SAMtools (e.g., the *phred*-scaled quality score for the variant allele must be higher than a certain value). Since the VCF also reports some additional information about the called SNPs, such as strand bias, quality by depth (coverage), mapping quality, read depth, and genotype quality that represents the quality of the called SNPs, users can further filter the called SNPs based on the cutoff values they choose for these metrics. Although SOAPsnp does not particularly use any internal filtering, it does provide several metrics in the output for each called SNP, e.g., consensus score, quality of best allele, quality of second best allele, and sequence depth. These metrics can be used as customized post-output filters.

### Part II Dataset

**Figure 1:**
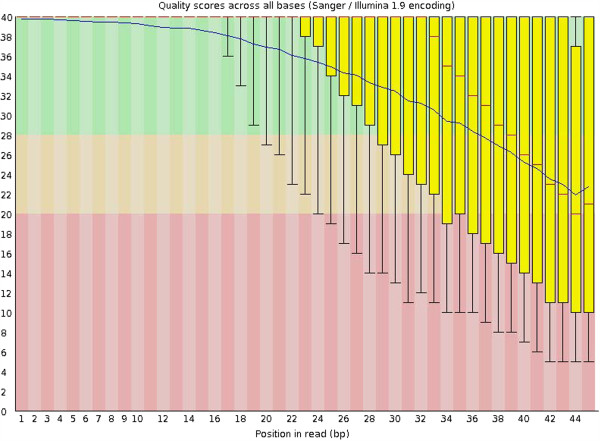
**Box plots for sequencing quality score (generated by FastQC).** The blue line represents the mean quality score for each base. Red lines represent medians. Yellow boxes represent 25^th^ to 75^th^ percentiles. The upper and lower whiskers represent 10 and 90 percentiles, respectively.

To study the performance of these different SNP calling tools in low-coverage data, we use a low-coverage (1-2X) whole-genome sequencing dataset from the pilot 1 of 1000 genome project: ERR000044. This dataset is sequenced from the sample #NA18550, with 6,333,357 45-bp-long reads generated. We first explore the sequencing quality by plotting the per-base quality scores using FastQC [42]. The sequence quality stays high at the beginning of the reads, and then drops quickly when reaching to the 3′ end of the reads (Figure 1).

### Part III SNP detection and comparison

**Figure 2:**
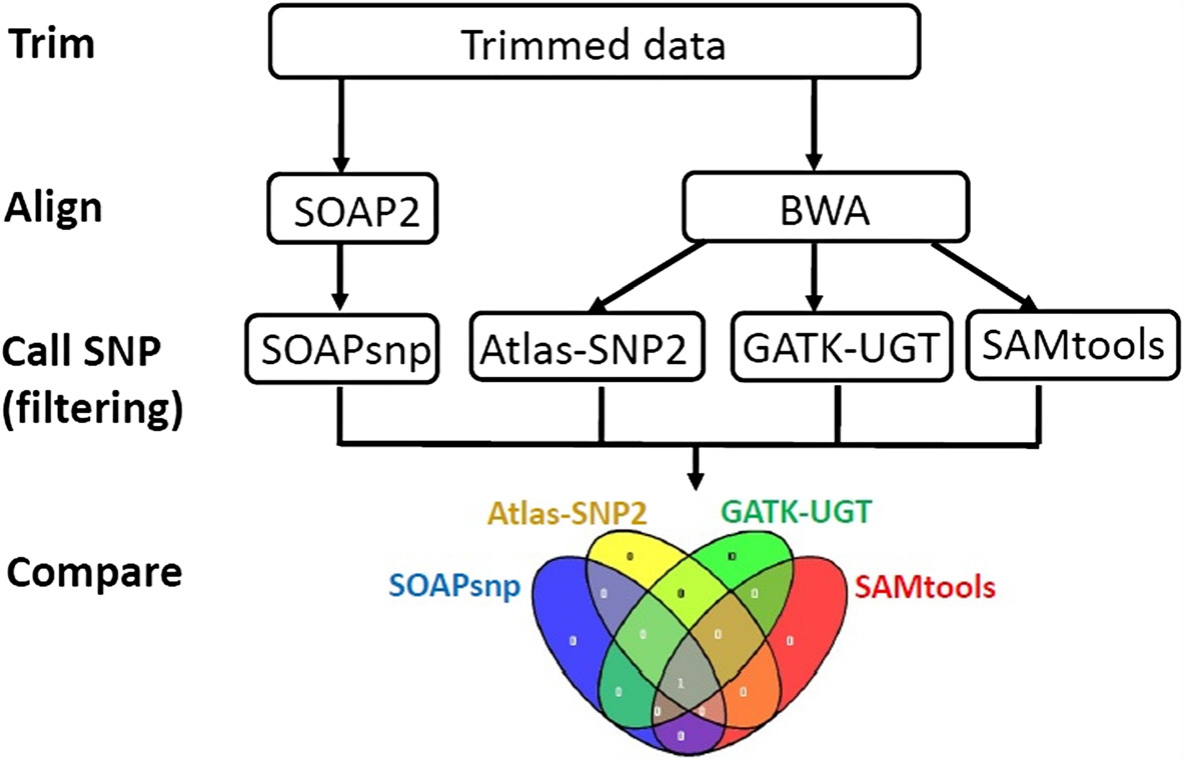
The overall workflow of comparing the four SNP calling algorithms.

There are four major steps in the overall workflow (Figure 2). First, before alignment, we trim off the low quality ends of reads using the trim function in the BRAT package [43]. In particular, the BRAT trim function is set to cut from both the 5′ and 3′ ends until it reaches bases with a quality score higher than 20 (i.e., 1% error rate). This trim function allows at most two Ns in each read. Second, alignments are conducted by either SOAP2 (version 2.21) or BWA (version 0.6.2), using the human genome 18 as the reference. At most two mismatches are allowed for each read, and only the reads aligned to unique positions are reported in the output files. Third, SNPs are called on chromosomes 1 and 2. All SOAPsnp callings are performed on SOAP2 alignment results, since SOAP2 is the only input format SOAPsnp can take. Because Atlas-SNP2, SAMtools, and GATK-UGT all require alignment results in the SAM format, which can be generated by BWA but not SOAP2, these three are performed on BWA alignment outputs. For the results of each SNP calling algorithm, we identify the dbSNPs and non-dbSNPs, using the dbSNP information (dbSNP build 130) downloaded from the UCSC Genome Browser [44]. Finally, we compare the SNP calling results from the four algorithms. Since Atlas-SNP2 requires at least 3X coverage to detect a variance, for a fair comparison, we only use SNPs with at least 3X in each algorithm. All detected SNVs are assigned to the following classes:

I. Single nucleotide variants (SNV) identified by only one SNP calling algorithm.

II. SNVs identified by any two SNP calling algorithms.

III. SNVs identified by any three SNP calling algorithms.

IV. SNVs identified by all four SNP calling algorithms.

This procedure is first conducted without any post-output filters. Then we apply filters based on the key metrics in the output of each SNP calling algorithm (Table 4), with different coverage cutoff values.

**Table 4:** Key metrics in each of the four algorithms

	**Metrics**
**SOAPsnp**	Consensus score [0, 99]
**Atlas-SNP2**	Posterior Probability
**SAMtools**	Genotype quality [0,99], QUAL
**GATK-UGT**	Genotype quality [0,99], QUAL, FisherStrand, HaplotypeScore, MappingQualityRankSumTest, ReadPosRankSumTest

## Results

### Part I Alignment and the impact of trimming

In raw data, among the 6,333,357 single-end reads, about 70% are aligned against human genome 18 by SOAP2 and BWA. 110–400 non-dbSNPs (potentially novel SNVs) are detected in each of the four SNP calling algorithms on chromosome 1 and 2 (Table 5A). Since trimming can remove low-quality bases and thus improve the alignment results [45], we trim the data using the trim function of the BRAT package. This process not only cuts off the low quality bases from both ends, but also discards reads that are shorter than 24-bp after trimming. As a result, 6,000 (0.1%) reads are removed. With slightly fewer reads (6,327,430) available, however, the number of aligned reads is increased by 100,000 (2%). Consequently, more SNPs are detected in trimmed data compared to raw data (Table 5B). Among the four algorithms, SOAPsnp calls more SNVs than the other three, in both raw and trimmed data. This is probably due to the fact that SOAPsnp has almost no internal filtering criterion after calling a SNV, meaning that it is not as stringent as the others. Although SOAP2 aligns slightly more reads than BWA, our previous study has shown that SOAP2 and BWA have similar alignment performance in trimmed data [45]. Therefore, the difference between SOAPsnp and the other three algorithms is less likely caused by alignment disagreements. When compared to SOAPsnp, Atlas-SNP2 calls significantly less SNVs than the other programs. The possible reasons are: 1) more stringent internal criteria are applied to determine SNVs, including coverage for variant alleles on both strands and the percentage of variant reads; 2) the threshold for posterior probability is set as ≥ 0.95. Since Atlas-SNP2 requires at least 3X coverage to call a SNV, we only report the called SNVs with ≥ 3X coverage in the other three algorithms. Without any coverage filtering (≥ 1X) in both raw and trimmed datasets, SOAPsnp calls dramatically more SNVs (about 4000) than SAMtools and GATK-UGT (about 2000). Since SNVs from raw and trimmed data show similar patterns, and trimmed data has more SNVs called, we use the trimmed data in further analysis.

**Table 5:** Number of SNVs called by each of the four algorithms using raw and trimmed data

**A. In raw data**			
	≥ 3X*	**dbSNPs**	**Non-dbSNPs**
**SOAPsnp**	940	545	395
**Atlas-SNP2**	432	315	117
**SAMtools**	532	376	156
**GATK-UGT**	669	444	225
**B. In trimmed data**			
	≥ 3X*	**dbSNPs**	**Non-dbSNPs**
**SOAPsnp**	968	564	404
**Atlas-SNP2**	448	321	127
**SAMtools**	570	398	172
**GATK-UGT**	729	478	251

* Atlas-SNP2 requires at least 3X to call a SNV. For the other three algorithms, we choose the called SNVs with ≥ 3X coverage.

### Part II Comparison without any filtering

**Figure 3:**
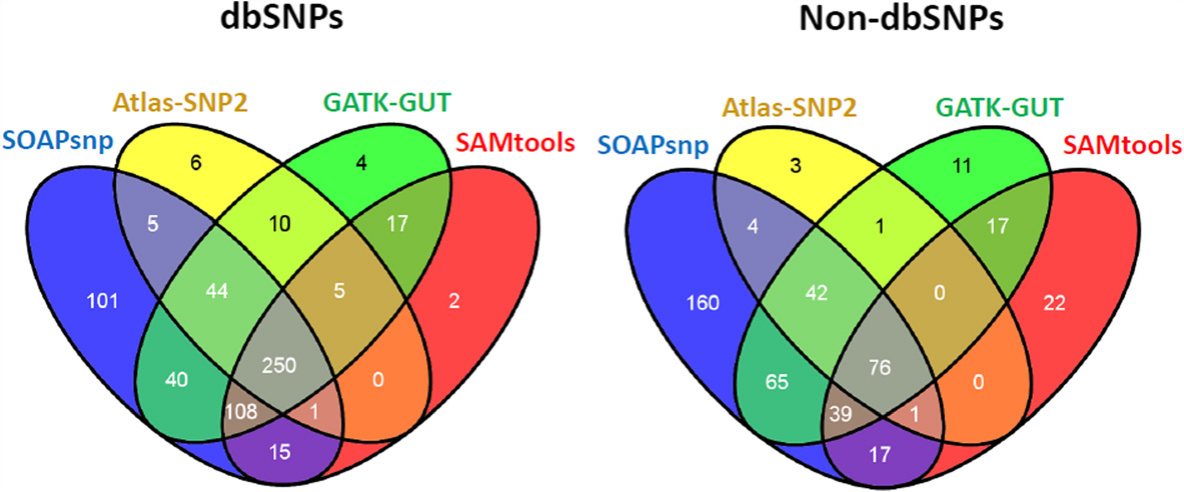
**The comparison results of trimmed data without any post-output filters.** All SNVs require ≥ 3X coverage.

In order to examine the agreement between the four algorithms, we compare both dbSNP and non-dbSNP results in trimmed data (see Figure 3). Overall, dbSNPs exhibit a better agreement than non-dbSNPs. This observation is consistent with our expectations, since the known dbSNP positions are more common and therefore more likely to be called. However, in terms of the performance of the four algorithms, dbSNPs and non-dbSNPs show similar patterns. Figure 3 shows that GATK-UGT and SAMtools have a better agreement compared to the other comparison pairs. This is probably due to one or more of the following reasons: 1) they are both Bayesian-based algorithms; 2) they incorporate similar information when determining the genotypes; and 3) they apply similar internal filters to the called SNVs. Because Atlas-SNP2 is more stringent than the other three calling programs, most of the SNVs called by Atlas-SNP2 are also called by at least one of other programs. Different from Atlas-SNP2, there are 101 dbSNPs and 160 non-dbSNPs that are only called by SOAPsnp. In order to investigate the difference between these SNVs that are only called by SOAPsnp and those that are also called by at least one of the other three algorithms, we compare their key metrics from the SOAPsnp output: consensus score, quality of best allele, quality of second best allele, and sequencing depth. No obvious difference is discovered between the two types of SNVs. Most of SNVs have a consensus score between 2 and 20, with only a few reaching the upper limit of 99. Moreover, most of SNVs are covered by 3 to 10 reads in total.

### Part III Exploration of key metrics in four SNP calling algorithms

#### *Key metrics in SOAPsnp*

We have examined SOAPsnp’s SNP calling quality in low-coverage data by checking the coverage and consensus scores for called dbSNPs and non-dbSNPs. We have found that the low coverage is often associated with low consensus scores, while the high coverage is often associated with high consensus scores. The consensus score in SOAPsnp represents how confident the algorithm is in calling a SNV. A higher value corresponds to a higher confidence. Therefore, using the consensus score as a filter is necessary in order to have accurate SNP calling in SOAPsnp. We have checked the distribution of consensus score in SOAPsnp results and have chosen filtering criteria based on this distribution. Table 6 shows that 91 SNVs have a consensus score < 5, indicating lower confidence. With a filtering criterion for consensus scores set at ≥ 5, 91 SNVs are removed and 877 SNVs are left in total.

**Table 6:** Number of SNVs called by the SOAPsnp with different cutoffs of consensus score

**Cutoffs**	**SNVs**	**dbSNPs**	**Non-dbSNPs**	SNVs ≥ *
**=0**	41	10	31	968
**=1**	8	2	6	927
**=2**	11	5	6	919
**=3**	6	2	4	908
**=4**	25	8	17	902
**=5**	261	179	82	877
**=6**	125	101	24	616
**=7**	21	13	8	491
**=8**	58	36	22	470
**=9**	24	12	12	412
**=10**	13	4	9	388

* number of SNVs that have consensus score ≥ the cutoff values.

#### *Key metrics in Atlas-SNP2*

Unlike SOAPsnp, Atlas-SNP2 provides a posterior probability for every potential SNV. It requires the users to set a threshold for the posterior probability. With a low coverage, many potential SNVs reported by Atlas-SNP2 have low posterior probabilities. In our previous analysis, we use “posterior probability ≥ 0.95” as a criterion to call SNVs, resulting in a much smaller number of SNVs when compared to the other three calling programs. In order to investigate whether posterior probability is a potential filter criterion, we set the cutoffs at ≥ 0.3 and then ≥ 0.1. With a lower threshold of 0.1, the number of SNVs called by Atlas-SNP2 increases from 448 to 539 (Table 7).

**Table 7:** Number of SNVs called by Atlas-SNP2 with different cutoffs of the posterior probability

**Posterior probability**	**SNVs**	**dbSNPs**	**Non-dbSNPs**
**≥ 0.95 (original setting)**	448	321	127
**≥ 0.3**	476	342	134
**≥ 0.1**	539	393	146

#### *Key metrics in GATK-UGT*

In the GATK-UGT output, there are several metrics associated with the quality of potential SNVs. We have checked a few important ones among them: “genotype quality”, “QUAL”, ‘FisherStrand”, “HaplotypeScore”, “MappingQualityRankSumTest”, and “ReadPosRankSumTest”.

1) “Genotype quality” represents the quality of the called SNVs. It ranges from 0 to 99, with higher values corresponding to higher qualities. To better understand the calling quality of GATK-UGT in low-coverage data, we have checked the distribution of the genotype quality. In this low-coverage dataset, for dbSNPs, the genotype ranges from 4 to 99, and 80% of dbSNPs have a genotype quality lower than 30; while for non-dbSNPs, the genotype ranges from 2 to 99, and 70% of non-dbSNPs have a genotype quality lower than 30. Then based on the distribution, we choose several different cutoff values for genotype quality, ≥ 5, 6, 7, 8, 9, and 10 (Table 8). With the cutoff set at ≥ 9, 53 SNVs (32 dbSNPs and 21 non-dbSNPs respectively) are removed, resulting in 676 remaining SNVs.

2) In VCF output, there is a metric called “QUAL”, a *phred*-scaled quality probability of the SNVs being a homozygous reference. A higher “QUAL” score indicates a higher confidence. In our dataset, all called SNVs have a QUAL value ≥ 30, which is a commonly used criterion for reliable SNP calling in GATK-UGT.

3) Another indicator of SNVs’ quality is strand bias, which looks for the instance where the variant allele is disproportionately represented on one strand. In GATK-UGT output, “FisherStrand” is a *phred*-scaled p-value using Fisher’s Exact test to detect strand bias. A higher “FisherStrand” value represents a more pronounced bias, indicating a false positive. The commonly used criterion for reliable SNV calling is to remove any SNV with a “FisherStrand” value > 60. In our dataset, the “FisherStrand” value for all SNVs ranges from 0 to 25. Therefore, there is no need for filtering using “FisherStrand”.

4) “HaplotypeScore” in GATK-UGT output is a measure of how well the data from a 10-base window around the called SNV can be explained by at most two haplotypes. Usually, with the instance of mismapped reads, there are more than two haplotypes around the SNV and this SNV is likely to be a false positive. A higher “HaplotypeScore” value represents a higher probability that the called SNV is artificial due to mismapping. In Table 9, we check the distribution of “HaplotypeScore” in dbSNPs and non-dbSNPs. The majority of SNVs have a low “HaplotypeScore” (≤ 10), indicating a generally good mapping in this dataset. Since the commonly used criterion for reliable SNVs calling is removing any SNV with a “HaplotypeScore” > 13, we use 13 as a filtering criterion, which removes 26 SNVs in total.

5) “MappingQualityRankSumTest” is a Wilcoxon rank test that tests the hypothesis that the reads carrying the variant allele have a consistently lower mapping quality than the reads with the reference allele. This metric is only available for the SNVs where both the variant allele and reference allele are supported by reads. In our dataset, there are 225 SNVs (97 dbSNPs and 126 non-dbSNPs) that have “MappingQualityRankSumTest” values, indicating that they have coverage in both variant and reference allele. In these 225 SNVs, the “MappingQualityRankSumTest” value ranges from−7 to 2 for dbSNPs, and−5 to 2 for non-dbSNPs. The commonly used criterion for reliable SNVs calling removes any SNV with a “MappingQualityRankSumTest” value < −12.5. Since in our dataset all SNVs are > −12.5, there is no need to apply any filter on the “MappingQualityRankSumTest” values.

6) “ReadPosRankSumTest” is a Mann–Whitney Rank Sum Test that tests the hypothesis that instead of being randomly distributed over the read, the variant allele is consistently found more often at the beginning or the end of a sequencing read. Similar to the “MappingQualityRankSumTest”, this metric is also only available for the SNVs where both the variant allele and reference allele are supported by reads. In our dataset, for the SNVs that actually have the “ReadPosRankSumTest” report, their values range from−5 to 6. These values satisfy the common criterion that the “ReadPosRankSumTest” value is ≥−20.

**Table 8:** Number of SNVs called by GATK-UGT with different cutoffs of genotype quality

**Cutoffs**	**SNVs**	**dbSNPs**	**Non-dbSNPs**
**≥ 0**	729	478	251
**≥ 5**	724	476	248
**≥ 6**	723	476	247
**≥ 7**	681	450	231
**≥ 8**	681	450	231
**≥ 9**	676	446	230
**≥ 10**	476	217	259

**Table 9:** Number of SNVs called by GATK-UGT with different cutoffs of HaplotypeScore

**Cutoffs**	**SNVs**	**dbSNPs**	**Non-dbSNPs**
**=0**	613	419	194
**≥ 1**	638	431	207
**≥ 2**	653	437	216
**≥ 5**	680	448	232
**≥ 10**	693	453	240
**≥ 13**	703	459	244
**≥ 20**	707	462	245
**≥ 30**	718	468	250
**all**	729	478	251

Based on the above exploration of the six key metrics in GATK-UGT output, we set a series of filtering criteria for reliable SNP calling by GATK-UGT: “genotype quality” ≥ 9; “QUAL” ≥ 30; “FisherStrand” ≤ 60; “HaplotypeScore” ≤ 13; “MappingQualityRankSumTest” ≥ −12.5; “ReadPosRankSumTest” ≥ −20. As a result, 650 SNVs (out of 729 raw SNVs) pass the filtering, with 427 dbSNPs and 223 non-dbSNPs. We will use this set of SNVs in a later analysis. Since “QUAL”, “FisherStrand”, “MappingQualityRankSumTest”, and “ReadPosRankSumTest” values all satisfy the criteria in our dataset, we cannot remove any SNV by applying filtering on these four metrics. However, they are all important metrics that are related to SNP quality. Thus, we recommend that users filter raw SNP calling results based on their values.

#### *Key metrics in SAMtools*

Similar to GATK-UGT, SAMtools reports the VCF output. We have checked two important metrics in SAMtools results: “genotype quality” and “QUAL”. In both dbSNPs and non-dbSNPs, the values of genotype quality range from 4 to 99. Setting different cutoff values for “genotype quality” does not filter out significantly more of the called SNP (Table 10). For “QUAL”, all SNVs have a QUAL value ≥ 3, which is a commonly used criterion for “QUAL” in SAMtools results. Therefore, for our dataset we do not apply any filter on SAMtools results and use the raw SNVs for a later analysis.

**Table 10:** Number of SNVs called by SAMtools with different cutoffs of genotype quality

**Cutoffs**	**SNVs**	**dbSNPs**	**Non-dbSNPs**
**≥ 4 (all)**	570	398	172
**≥ 5**	567	397	170
**≥ 6**	565	396	169
**≥ 7**	564	395	169
**≥ 8**	563	395	168
**≥ 9**	559	393	166
**≥ 10**	558	393	165

### Part IV Comparison with filtering using key metrics and coverage

To compare the four algorithms under different coverage levels, we use the SNP calling results with filtering criteria applied in each calling program, and then add the filtering of coverage with several cutoff values, ≥ 4X, 5X, 6X, 7X, 8X, 9X, and 10X (Table 11). The number of SNVs called by each calling program decreases dramatically by more than 50% when the cutoff increases from 3X to 4X, and drops to about 15% at 10X. With 3X, SOAPsnp calls more SNVs than the other calling programs, while Atlas-SNPs calls the least. However, when the coverage cutoff increases, the number of SNVs called by each calling program becomes more similar, with SOAPsnp calling slightly more.

**Table 11:** Number of SNVs called by each of the four algorithms with different coverage cutoffs

**Coverage cutoffs**	**SOAPsnp**	**Atlas-SNP2**	**GATK-UGT**	**SAMtools**
**≥ 3X**	877 (537, 340)	539 (393, 146)	650 (427, 223)	570 (398, 172)
**≥ 4X**	397 (230, 167)	291 (195, 96)	309 (187, 122)	270 (174, 96)
**≥ 5X**	280 (162, 118 )	218 (138, 80)	223 (127, 96)	203 (121, 82)
**≥ 6X**	222 (130, 92)	187 (116, 71)	186 (105, 81)	167 (100, 67)
**≥ 7X**	194 (115, 79)	160 (99, 61)	156 (93, 63)	145 (87, 58)
**≥ 8X**	168 (99, 69)	145 (93, 52)	134 (81, 53)	127 (81, 46)
**≥ 9X**	153 (88, 65)	138 (87, 51)	126 (75, 51)	115 (73, 42)
**≥ 10X**	137 (78, 59)	126 (82, 44)	111 (65, 46)	100 (64, 36)

**Figure 4:**
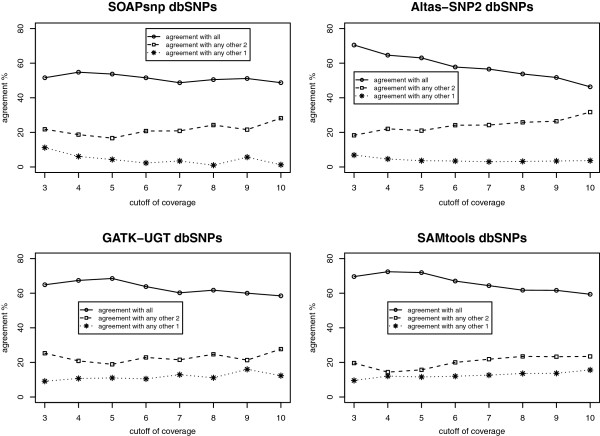
The agreement of dbSNPs with different coverage cutoffs in each of the four algorithms.

**Figure 5:**
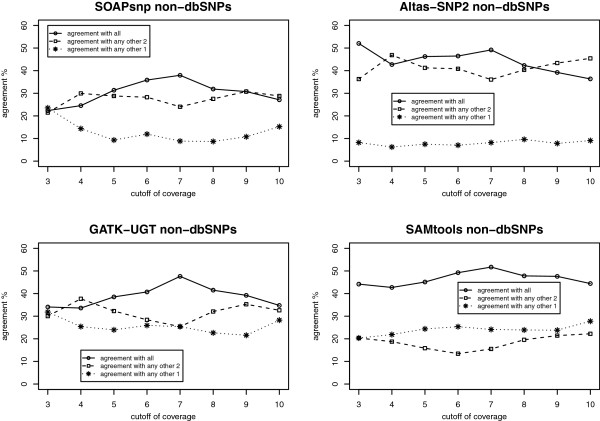
The agreement of non-dbSNPs with different coverage cutoffs in each of the four algorithms.

Table 11 shows the changing patterns of the number of SNVs as the coverage cutoff level increases. Although the numbers of SNVs identified by the different calling programs become more similar as the coverage cutoff increases, it is unclear whether the agreement of different calling programs and their performance will increase accordingly. In order to address this question, we have done further comparisons using the following two methods: Method 1 checks the agreement among different calling algorithms (see Table 12, Figures 4 and 5), and Method 2 calculates empirical positive calling rates and sensitivities (see Table 13). For both methods, we check dbSNPs and non-dbSNPs separately.

**Table 12:** Comparing four algorithms using different coverage cutoffs for dbSNPs and non-dbSNPs

**A. dbSNPs**					
**Coverage cutoffs**	**Total**	**By 1**	**By 2**	**By 3**	**By 4**
**≥ 3X**	592	108 (18.24%)	82 (13.85%)	125 (21.11%)	277 (46.79%)
**≥ 4X**	276	68 (24.64%)	32 (11.59%)	50 (18.12%)	126 (45.65%)
**≥ 5X**	201	61 (30.35%)	20 (9.95%)	33 (16.42%)	87 (43.28%)
**≥ 6X**	169	54 (31.95%)	15 (8.88%)	33 (19.53%)	67 (39.64%)
**≥ 7X**	153	53 (34.64%)	15 (9.80%)	29 (18.95%)	56 (36.60%)
**≥ 8X**	134	43 (32.09%)	12 (8.96%)	29 (21.64%)	50 (37.31%)
**≥ 9X**	123	38 (30.89%)	15 (12.20%)	25 (20.33%)	45 (36.59%)
**≥ 10X**	110	34 (30.91%)	11 (10.00%)	27 (24.55%)	38 (34.55%)
**B. non-dbSNPs**					
**Coverage cutoffs**	**Total**	**By 1**	**By 2**	**By 3**	**By 4**
**≥ 3X**	402	151 (37.56%)	99 (24.63%)	76 (18.91%)	76 (18.91%)
**≥ 4X**	211	76 (36.02%)	41 (19.43%)	53 (25.12%)	41 (19.43%)
**≥ 5X**	161	57 (35.04%)	30 (18.63%)	37 (22.98%)	37 (22.98%)
**≥ 6X**	127	38 (29.92%)	27 (21.26%)	29 (22.83%)	33 (25.98%)
**≥ 7X**	106	33 (31.13%)	21 (19.81%)	22 (20.75%)	30 (28.30%)
**≥ 8X**	93	32 (34.41%)	17 (18.28%)	22 (23.66%)	22 (23.66%)
**≥ 9X**	87	28 (32.18%)	16 (18.39%)	23 (26.44%)	20 (22.99%)
**≥ 10X**	79	25 (31.65%)	18 (22.78%)	20 (25.32%)	16 (20.25%)

“Total” means the total number of SNVs called by four algorithms. “By 1” means the number (percentage) of SNVs called by only one of the four algorithms. “By 2” means the number (percentage) of SNVs called by any two algorithms. “By 3” means the number (percentage) of SNVs called by any three algorithms. “By 4” means the number (percentage) of SNVs called by four algorithms.

**Table 13:** Positive calling rate and sensitivity

		**Empirical truth**	
		**SNV**	**Not SNV**
**Program’s calling results**	called as SNV	A	B
	called as Reference (i.e., not SNV)	C	--

For a specific calling program (e.g., SOAPsnp), A is the number of SNVs identified as an empirical truth (i.e., called by at least 3 calling programs) and also called by this calling program; B is the number of SNVs identified as an empirical truth, but not called by this calling program; C is the number of SNVs called by this calling program, but is not an empirical truth. Positive calling rate is calculated as A/(A + B); sensitivity is calculated as A/(A + C).

#### *Method 1: check the agreement among different calling programs*

For dbSNPs, using the original setting (≥ 3X), there are 592 unique dbSNPs called by the four algorithms, and 46.79% of them are common among all the calling programs. When increasing the cutoff of coverage to 4X, although the number of unique dbSNPs drops dramatically from 592 to 276, the percentage of agreements among the four calling programs remains similar (Table 12A). With a further increase of coverage cutoff values, the number of unique dbSNPs continuously decreases, while the agreements stay similar (Table 12A). For each SNP calling program, we plot the agreement with other algorithms under different coverage cutoffs (Figure 4). For SOAPsnp, even though the number of called dbSNPs drops dramatically, the agreements with other calling programs do not change as much as the coverage cutoff increases. For Atlas-SNP2, the percentage of agreement with the other three calling programs decreases when the coverage cutoff increases. This is probably due to the fact that with a lower cutoff (≥ 3X), Atlas-SNP2 calls much fewer than the other calling programs. Therefore, compared to other programs, the 277 agreement dbSNPs take a larger portion among all SNVs called by Atlas-SNP2. However, when the coverage cutoff increases, the number of dbSNPs called by Atlas-SNP2 is far more similar to the other algorithms, therefore the percentage of agreement in Atlas-SNP2 becomes smaller than ≥ 3X. Compared to SOAPsnp and Atlas-SNP2, GATK-UGT and SAMtools exhibit a higher agreement with other calling programs. 60-70% of their dbSNPs are called by all four programs, 20% are called by three programs, and about 10% are called by two programs (see Figure 4 bottom panel). Moreover, in both GATK-UGT and SAMtools, when the cutoff increases from 3X to 5X, the percentage of dbSNPs called by all four programs increases 3-4%.

For non-dbSNPs, the comparison results show similar patterns as dbSNPs, but with a lower percentage of agreement (Table 12B). The number of unique non-dbSNPs called by the four algorithms drops from 402 to 211 when the coverage cutoff increases from 3X to 4X, and finally decreases to 79 when the coverage cutoff is 10X. The percentage of non-dbSNPs called by all four calling programs increases over the different coverage cutoffs, especially from 3X to 7X. While the percentage of non-dbSNPs only called by one algorithm decreases over the cutoffs, from 37.56% in 3X to 31.65% in 10X. For each calling program, we plot the agreement with other algorithms under different coverage cutoffs (Figure 5). Among the four calling algorithms, SOAPsnp shows the lowest percentage of agreements with others. These low agreements are probably due to the fact that SOAPsnp always calls more SNVs than other programs under different coverage levels. In all four calling programs, the percentage of agreements increases over the coverage cutoff values, especially from 3X to 7X, indicating that filtering the non-dbSNPs with a higher coverage threshold improves the agreement among the four algorithms.

#### *Method 2: calculate empirical positive calling rates and sensitivity*

For this comparison method, we choose the variants that are called by at least three calling programs as the “empirical truth”, and then investigate the calling performance of each SNP calling program based on this empirical truth by calculating both the positive calling rate and the sensitivity. We then compare the four calling programs at different coverage levels using these rates. The positive calling rate and the sensitivity are calculated as Positive calling rate = A/(A + B), and Sensitivity = A/(A + C) as shown in Table 13. In these formulas, A is the number of SNVs identified as an empirical truth (i.e., called by at least 3 calling programs) and also called by this calling program; B is the number of SNVs identified as an empirical truth, but not called by this calling program; and C is the number of SNVs called by this calling program, but is not an empirical truth.

The results of comparing four SNP calling algorithms using the empirical positive calling rate and sensitivity are shown in Table 14 and Table 15 and are explained below.

1) For calling dbSNP positions, Table 14A (dbSNPs) shows that SOAPsnp has a relatively lower positive calling rate. This is because SOAPsnp tends to call more variants than the other three calling programs, suggesting a higher false positive rate. GATK has a relatively higher positive calling rate than the others at different coverage levels for calling dbSNPs. Atlas-SNP2 and SAMtools tend to stay between SOAPsnp and GATK.

2) For calling non-dbSNP positions, similar to dbSNPs, Table 14B shows that SOAPsnp tends to call more false positive variants since it lacks stringent internal filtering criteria. Atlas-SNP2 shows the highest positive calling rate. This is probably because it is the most stringent calling program. GATK has a higher positive calling rate than SOAPsnp and SAMtools.

3) As far as the positive calling rate is concerned, Atlas-SNP2 and GATK perform better than SOAPsnp and SAMtools on both dbSNPs and non-dbSNPs. With the change of coverage level, the comparison results are relatively stable.

4) For calling dbSNPs and non-dbSNPs, Table 15 shows that, with the exception of SAMtools, the other three programs all have very high sensitivity in calling SNVs. Overall the sensitivity of all calling programs are pretty stable across the different coverage levels, except that Atlas-SNP2’s sensitivity is a bit low at 3X coverage.

**Table 14:** Positive calling rates of the four calling programs under different coverage cutoffs for dbSNPs and non-dbSNPs

**A. dbSNPs**				
**Coverage cutoffs**	**SOAPsnp**	**Atlas-SNP2**	**GATK-UGT**	**SAMtools**
≥ 3X	0.734	0.888	0.902	0.892
≥ 4X	0.735	0.867	0.882	0.868
≥ 5X	0.704	0.841	0.874	0.876
≥ 6X	0.723	0.819	0.867	0.870
≥ 7X	0.696	0.808	0.817	0.862
≥ 8X	0.747	0.796	0.864	0.852
≥ 9X	0.727	0.782	0.813	0.849
≥ 10X	0.769	0.780	0.862	0.828
**B. non-dbSNPs**				
**Coverage cutoffs**	**SOAPsnp**	**Atlas-SNP2**	**GATK-UGT**	**SAMtools**
≥ 3X	0.438	0.863	0.628	0.628
≥ 4X	0.545	0.896	0.713	0.615
≥ 5X	0.602	0.875	0.708	0.610
≥ 6X	0.641	0.873	0.691	0.627
≥ 7X	0.620	0.852	0.730	0.672
≥ 8X	0.594	0.827	0.736	0.674
≥ 9X	0.615	0.824	0.745	0.690
≥ 10X	0.559	0.818	0.674	0.667

**Table 15:** Sensitivities of the four calling programs under different coverage cutoffs for dbSNPs and non-dbSNPs

**A. dbSNPs**				
**Coverage cutoffs**	**SOAPsnp**	**Atlas-SNP2**	**GATK-UGT**	**SAMtools**
≥ 3X	0.980	0.868	0.958	0.883
≥ 4X	0.960	0.960	0.938	0.858
≥ 5X	0.950	0.967	0.925	0.883
≥ 6X	0.940	0.950	0.910	0.870
≥ 7X	0.941	0.941	0.894	0.882
≥ 8X	0.937	0.937	0.886	0.873
≥ 9X	0.914	0.971	0.871	0.886
≥ 10X	0.923	0.985	0.862	0.815
**B. non-dbSNPs**				
**Coverage cutoffs**	**SOAPsnp**	**Atlas-SNP2**	**GATK-UGT**	**SAMtools**
≥ 3X	0.912	0.546	0.837	0.570
≥ 4X	0.968	0.915	0.926	0.628
≥ 5X	0.959	0.946	0.919	0.676
≥ 6X	0.952	1.000	0.903	0.677
≥ 7X	0.942	1.000	0.885	0.750
≥ 8X	0.932	0.977	0.886	0.705
≥ 9X	0.930	0.977	0.884	0.674
≥ 10X	0.917	1.000	0.861	0.667

## Discussion

Identifying a reliable list of SNPs is critical when analyzing NGS data. For data with high-coverage and/or multiple samples, previous studies have shown that different SNP calling algorithms have a good agreement between each other and have high true positive rates [36,39,40]. However, for single-sample low-coverage data, it is difficult to call SNVs with high confidence. In order to provide insights into the choice of SNP calling programs, we have compared the performance of four commonly used SNP calling algorithms using low coverage sequencing data.

### About the four SNP calling algorithms and their post-output filtering

Out of the four algorithms, SOAPsnp calls many more SNVs compared to the others. This is probably because it has less internal filtering criteria. After applying the criterion that removes any SNVs with a consensus score lower than 5, the total number of SNVs called by SOAPsnp decreases and becomes more similar to the other algorithms. In the SOAPsnp output file, the consensus score is an important metric representing the quality of calling a SNP. Therefore, when processing low-coverage data, we recommend that users apply the consensus score as a post-output filter for SOAPsnp results.

Atlas-SNP2 is much more stringent compared to the other three algorithms. 97% of the SNVs called by Atlas-SNP2 are also called by at least one of the other three calling programs. With a much lower threshold for posterior probability, Atlas-SNP2 calls more SNVs but still fewer than the other algorithms. Since it has the lowest number of called SNVs, Atlas-SNP2 appears to have a higher positive calling rate and sensitivity when compared to the other calling programs (Tables 14 and 15). However, when using Atlas-SNP2 to deal with low-coverage dataset, the users should be careful with the filtering settings. For example, in this study, we set the threshold for posterior probability at 0.1, which indicates a low confidence in calling a SNP. Because Atlas-SNP2 is much more stringent than the other programs, even with a low posterior probability, the called SNVs are still very likely to agree with other calling programs.

Compared to the above two algorithms, GATK-UGT and SAMtools call a moderate number of SNVs. When using the GATK-UGT package, applying the common criteria is necessary, including “Genotype quality”, “QUAL”, “MappingQualityRankSumTest”, “FisherStrand”, “HaplotypeScrore”, and “ReadPosRankSumTest”. With the SAMtools program, filtering out the SNVs with low genotype quality and low “QUAL” value can help improve the accuracy in SNP calling.

Filtering out the low quality SNVs is an important step before performing further analysis, especially for low-coverage data. When choosing the criteria for filtering, it is important not only to consider the commonly used standards, but also to take into account the characteristics of each specific dataset. For example, in our dataset, all the SNVs have little or no strand bias, have high “MappingQualityRankSumTest” scores, and have high “ReadPosRankSumTest” scores. Setting the threshold of genotype quality at 9 gives a similar number of SNVs compared to others. Besides the key metrics that we have explored in the Result section, each algorithm provides additional information. For instance, SOAPsnp reports the quality of variant and reference alleles, number of reads covering the variant and reference alleles, average copy number, and more. GATK-UGT and SAMtools both report their results in VCF, which can include many metrics. Users may check these metrics based on the characteristics of their own data if necessary, though we did not find these metrics to be very helpful (data not shown).

### About the impact of coverage

Coverage is an important factor to consider when assessing the quality of called SNVs. Without any coverage filtering (i.e., just ≥ 1X coverage), the results of the four calling programs can be dramatically different. Usually, high coverage regions or bases tend to have higher calling qualities (e.g., higher consensus scores in SOAPsnp, higher posterior probabilities in Atlas-SNP2, and higher genotype qualities in SAMtools and UGT). Low coverage regions or bases tend to have lower SNP calling qualities. However, there is not a simple linear relationship between coverage and the genotype quality scores that are generated by different SNP calling programs.

Our results show that when increasing the coverage levels for each calling program, the number of identified SNVs drops dramatically in all calling programs. However, increasing sequencing coverage cutoffs does not necessarily lead to an increase in agreement among the different calling programs. In fact, our comparison results show that the impact of coverage on calling agreement is small except that we see some agreement increase in non-dbSNPs when the coverage level changes from 3X to 7X. This may sound counter-intuitive. However, this observation can be explained by the fact that the four programs use different statistical methods and algorithms, which model different aspects of the sequencing information. These differences lead to the complex correlations of output metrics.

Filtering out many low-coverage SNVs may result in a sacrifice of missing novel SNVs. For example, the number of called SNVs in each calling program decreases by more than 50% when the coverage cutoff increases from 3X to 4X, and drops to 15% at 10X. Therefore, caution should be used when choosing coverage as a filtering criterion. Simply choosing the SNVs called with high coverage might not be sufficient. This is because, with a higher threshold of coverage, the users may over-filter the results and miss novel SNPs related to the disease of their interest.

### About the generalization of our results and decision making

In this paper, we use a set of single-end data, which is one mate of a pair-end dataset. We have also conducted the same analysis using a different single-end sequencing dataset and have arrived at the same conclusion. Therefore, we only report the results from the first dataset we used. In addition, the results we report here are generated by analyzing chromosomes 1 and 2 together. We have also analyzed chromosomes 1 and 2 separately and get the same conclusion as when they are combined. Furthermore, the findings in this paper are similar to the results reported by other researchers [46]. Therefore, our comparison methods and results can be generally applied to low-coverage sequencing data. In addition, although this paper mainly focuses on the SNP calling in a single sample, our methods and conclusion can be easily applied to the variant calling in multiple samples. In particular, the empirical-based positive calling rate and sensitivity analysis can serve as an empirical standard for comparing algorithms in multiple-sample SNP calling.

Overall, the four calling programs have very low agreement amongst each other, with only roughly 35% ~ 45% for dbSNPs and 19% ~28% for non-dbSNPs. For very low coverage data, it might be wise to choose a concordance among two or more SNP calling program instead of just using one algorithm. However, this may result in a high false-negative rate, with many true SNVs being missed. In addition, choosing filtering cutoff values for coverage and different quality scores with high and low values may have the same advantages and disadvantages as choosing a single SNP calling program vs. using the concordance of two or more SNP calling programs. Therefore, as far as the experimental validation of novel SNVs is concerned, we recommend that users employ a comprehensive strategy in their validation plan. First, in order to obtain a high experimental validation rate, the users may choose the SNVs that are called by more than one algorithm and with high metrics (e.g., coverage and quality scores) in the beginning of the validation process. Then, if the validation success rate is high, the users may validate more low coverage SNVs called by multiple calling programs, or SNVs called by only one program but with high quality. This approach can both ensure an effective validation and avoid missing many true disease-contributing SNVs.

## Conclusions

We have compared the performance of four SNP calling programs in a low-coverage single-sample sequencing dataset. It is important to filter out the SNVs of low quality using different metrics (e.g., quality scores and coverage). Our results show that the concordance among these different calling algorithms is low, especially in non-dbSNPs, and increasing the cutoff values of coverage has little effect on improving the concordance. This is probably due to discrepancies in the statistical methods and algorithms that these calling programs employ. Additionally, to provide an empirical standard for choosing a SNP calling program, we have calculated the empirical positive calling rate and sensitivity for each calling algorithm under different cutoffs of coverage. We have found that dbSNPs have generally higher rates compared to non-dbSNPs, suggesting lower quality in called non-dbSNPs in low-coverage sequencing data. Moreover, among the four calling programs, GATK and Atlas-SNP2 show a relatively higher positive calling rate and sensitivity when compared to the others, and GATK tends to call more SNVs than Atlas-SNP2. Therefore, if users intend to use only one calling program, we recommend GATK. However, in order to increase the overall accuracy, we advocate for employing more than one SNP calling algorithms.

## Competing interests

The authors declare that they have no competing interests.

## Authors’ contributions

Both authors developed and performed the statistical and computational analysis, drafted, revised, and approved the manuscript.
